# Exposure to Perfluoro-Octanoic Acid Associated With Upstream Uncoupling of the Insulin Signaling in Human Hepatocyte Cell Line

**DOI:** 10.3389/fendo.2021.632927

**Published:** 2021-09-03

**Authors:** Luca De Toni, Andrea Di Nisio, Maria Santa Rocca, Diego Guidolin, Alice Della Marina, Loris Bertazza, Stefania Sut, Edoardo Purpura, Micaela Pannella, Andrea Garolla, Carlo Foresta

**Affiliations:** ^1^Department of Medicine, Unit of Andrology and Reproduction Medicine, University of Padova, Padova, Italy; ^2^Department of Neuroscience, University of Padova, Padova, Italy; ^3^Endocrinology Unit, Department of Medicine (DIMED), University of Padova, Padova, Italy

**Keywords:** glycogen, membrane, glut-4, GM3, PDMP

## Abstract

Perfluoro–alkyl substances (PFAS) are chemical pollutants with prevalent stability and environmental persistence. Exposure to PFAS, particularly perfluoro-octanoic acid (PFOA), has been associated with increased diabetes-related cardiovascular mortality in subjects residing areas of high environmental contamination, however the exact pathogenic mechanism remains elusive. Here we used HepG2 cells, an *in vitro* model of human hepatocyte, to investigate the possible role of PFOA exposure in the alteration of hepatic glucose metabolism. HepG2 cells were exposed for 24 hours to PFOA at increasing concentration from 0 to 1000 ng/mL and then stimulated with 100 nm Insulin (Ins). The consequent effect on glycogen synthesis, glucose uptake and Glut-4 glucose transporter translocation was then evaluated by, respectively, Periodic Acid Schiff (PAS) staining, 2-deoxyglucose (2-DG) uptake assay and immunofluorescence. Exposure to PFOA was associated with reduced glycogen synthesis and glucose uptake, at concentration equal or greater than, respectively, 0,1 ng/mL and 10 ng/mL, with parallel impaired membrane translocation of Glut-4 upon Ins stimulation. Western blot analysis showed early uncoupling of Insulin Receptor (InsR) activation from the downstream Akt and GSK3 phosphorylation. Computational docking analysis disclosed the possible stabilizing effect of PFOA on the complex between InsR and GM3 ganglioside, previously shown to be associated with the low grade chronic inflammation-related insulin resistance. Consistently, long term treatment with glucosyl-ceramide synthase inhibitor PDMP was able to largely restore glycogen synthesis, glucose uptake and Glut-4 translocation upon Ins stimulation in HepG2 exposed to PFOA. Our data support a novel pathogenic mechanism linking exposure to PFOA to derangement of hepatocyte cell metabolism.

## Introduction

Environmental pollution by perfluoro–alkyl substances (PFAS) increasingly gained attention because of the several health issues related to their exposure (1). Concerns related with PFAS mainly rely on their high chemical inertia, physical stability and water repellency, resulting in environmental persistence and significant bioaccumulation through the trophic web bio-magnification (2). Importantly, results from toxicokinetic studies showed that the elimination of PFAS varies greatly across species and within genders. As an example, serum half-lives of the two PFAS of major environmental interest, such as perfluoro-octanoic acid (PFOA) and perfluoro-octane sulfonate (PFOS), show typical values in mice of 16 and 30 days in females and 22 and 43 days in males, respectively. In humans, the corresponding estimated half-life values for PFOA and PFOS range from 2,3 to 3,3 years in females and from 5,4 to 8,5 years in males, respectively (3). The evaluation of the specific health risks associated with the exposure to PFAS has received a considerable pulse from the identification of populations of individuals characterized by high blood levels of these chemical compounds for reasons concerning occupation or local environmental pollution. In a recent study, Girardi and Merler (3) evaluated the standardized mortality ratio in male employees of a chemical plant for the production of PFOA and PFOS, who had worked for at least six months in the period 1968-2009. In a final cohort of 462 subjects, the comparison between the reference mortality rates of the regional population and that of the neighboring metalworking plant, showed that employees of the chemical plant had an increased overall mortality for causes attributable to diabetes, liver cancer and liver cirrhosis. In addition, the relative risk of disease was associated with the probability of exposure to PFAS as determined by the tertiles of cumulative PFOA serum concentrations (4). Importantly, the production activities of the aforementioned plant has been associated with major issues of groundwater contamination by PFAS, resulting in serum concentrations of PFOA up to more than 1000 ng/mL in the population with background residential exposure (5). Accordingly, an increase in prevalence for diabetes mellitus, respectively of 17% in men and 14% in women, has been reported for people residing in the area of high environmental exposure to PFAS, and particularly, to PFOA [file:///F:/Luca/Pubblicazioni/PFOA%20Fegato/Biblio/Documento%20di%20Sintesi_23%2004%202018_def.pdf].

Previous studies on the bio-accumulation of PFAS in body tissues showed peculiar trends according to the organ evaluated. To this regard, Pérez et al. reported that the most prevalent accumulation of both PFOA and PFOS in humans takes place in bone and liver (6). Differently, *in vitro* data obtained by Sanchez Garcia et al. in fully differentiated 3T3-L1 mouse adipocytes, showed a detectable cell accumulation of PFOS with negligible or none accumulation for PFOA (7). This evidence confirmed a series of previous reports depicting a major liver toxicity of PFAS, largely independent from the route of access of these compounds, whether oral or transdermal (8–12). Despite this considerable amount of evidence, available data on the molecular mechanisms linking the exposure to PFAS with altered hepatocyte function are scattered, particularly the evaluation of the specific effects on liver energy metabolism remain under-investigated.

In this study we investigated the possible causal role of PFAS exposure in the alteration of hepatic glucose metabolism by the use of HepG2 cells, an *in vitro* model human hepatocyte. To this aim, we focused on PFOA, which represents the major chemical pollutant of the perfluorolkyl class in the Veneto region in northeastern Italy (13), and on the possible early impairment of the Insulin (Ins) signaling in hepatocyte based on acknowledged ability of PFAS to interfere with the biophysical properties of plasma membrane (13, 14).

## Materials and Methods

### Chemicals and Reagents

Perfluoro-octanoic acid native compound (Wellington Laboratories, Southgate, Ontario, Canada) and (±)-threo-1-Phenyl-2-decanoylamino-3-morpholino-1-propanol hydrochloride (PDMP) were dissolved in DMSO (both from Sigma-Aldrich S.r.l., Milan, Italy) to a stock concentration of 50 μg/mL and stored at -20°C until use. Protease Inhibitor Cocktail powder was also purchased from Sigma-Aldrich. Human recombinant Insulin (Ins) was purchased from SERVA Electrophoresis GmbH (Euroclone, Pero, Milan, Italy). Periodic Acid Schiff (PAS) staining kit and Colorimetric Glucose Uptake Assay Kit were both purchased from Abcam (Abcam-Prodotti Gianni, Milano, Italy). Rabbit anti-Insulin Receptor (InsR) β-chain, phospho-InsR β-chain (P-InsR), phospho-Akt (T308, P-Akt) and phospho-GSK-3β (P-GSK) were purchased from Cell Signaling Technologies (Euroclone). Rabbit anti-human total Akt (T-Akt, N3C2), rabbit anti-human total GSK-3β (T-GSK3, C1C3) were purchased from GeneTeX (CA, USA), rabbit-anti human GLUT4 was purchased from Invitrogen (Invitrogen-Thermo Scientific, Monza – Monza Brianza, Italy). Finally, mouse anti-GM3 ganglioside IgM and secondary reagent Goat Anti-Mouse IgM (H&L) antibody were purchased from Creative Biolabs Inc. (NY, USA).

### Cell Culture

Human hepatocellular carcinoma cell line HepG2 was a kind gift from Dr Santina Quarta (Department of Medicine, University of Padova, Padova, Italy). Cells were maintained in minimum essential medium (MEM), supplemented with 10% fetal bovine serum (FBS), antibiotics penicillin-G/strepromycin (Gibco-Thermo Fisher) and antifungal amphotericin B (Euroclone). Cells were propagated when 90% confluence was reached.

In stimulation experiments, cells were starved overnight in serum-free medium and then exposed to PFOA, at a concentration ranging from 0 ng/mL to 1000 ng/mL, diluted in MEM supplemented with 10% charcoal-treated FBS (Thermo Scientific). For glycogen synthesis assay, cells were stimulated for 3 hours with 100 nM Ins, rinsed with cold PBS, fixed with 4% Paraformaldehyde/PBS solution for 10 minutes at room temperature, and then stained for glycogen with PAS staining kit according to manufacturer’s procedure. Three representative fields for each experimental condition were captured by Nikon Eclipse TS100 inverted microscope equipped with DS-Fi1 camera (Nikon). Computer assisted image analysis was performed by the ImageJ image analysis software (15). Briefly, from the obtained grayscale image the integrated density was measured and then used as an index of staining intensity as reported as fold increase compared to untreated cells.

For the evaluation of glucose-uptake, cells were starved in serum free/glucose free Krebs-Ringer-HEPES buffer for 2 hours and then stimulated with 100 nM Ins for 20 minutes at 37°C in presence of 2-deoxyglucose (2-DG). Intracellular 2-DG content was then quantified with Glucose Uptake Assay Kit according to the manufacturer’s instructions.

### Immunofluorescence

Cells were cultured onto sterile coverslips of 13 mm diameter, treated with 0.5% gelatin. After the exposure to PFOA, as previously described, cells were subsequently stimulated with 100 nM Ins for 20 minutes at 37°C, washed with cold PBS and then fixed with 4% Paraformaldehyde/PBS solution for 10 minutes at room temperature. Cells were then permeabilized with 1% Triton X100/PBS solution for 10 minutes at room temperature and then saturated with 5% normal donkey serum/5% bovine serum albumin/PBS solution for 30 minutes at room temperature. Cells were then incubated with anti-Glut4 antibody (1:400) overnight at 4°C and the primary immunoreaction was detected with FITC-conjugated-goat-anti-rabbit IgG secondary reagent (1:200 Santa Cruz Biotechnology-D.B.A. Italia, Segrate, Milano, Italy). Cells were finally counterstained with 4′,6-diamidine-2′-phenylindole dihydrochloride (DAPI, Sigma-Aldrich). Samples on glass coverslips were mounted with anti-fade buffer on regular microscope slides and analyzed with video-confocal (VICO) fluorescence microscope (Nikon, Firenze, Italy).

For colocalization studies, approximately 2 × 10^7^ cells/mL HepG2 cells per condition were stained with rabbit anti-InsR β-chain IgG (Euroclone) and mouse anti-GM3 IgM (Creative Biolabs, NY, USA) followed by, respectively, Alexafluor 488-conjugated goat anti-rabbit IgG (Abcam) and Rhodamine-conjugated goat anti-mouse IgM antibodies (Creative Biolabs). Localization of InsR and GM3 antigens on cells was performed using the Amnis ImageStream Mk II Imaging flow cytometer (Luminex, Thermo Fisher Scientific). Co-localization of InsR and GM3 was assessed on single, focused cells that were positive for both fluorochromes. Data were acquired with INSPIRE software and analyzed using the IDEAS 6.3 analysis software (Luminex, Thermo Fisher Scientific). The degree of co-localization was calculated using the bright detail similarity feature, a pixel by pixel comparison of the two signals, which is based on the log transformed Pearson’s coefficient. Cells with a similarity score of 2.5 or higher were classified as co-localized.

### Western Blot

Cells exposed to PFOA were subsequently stimulated with 100 nM Ins for 20 minutes at 37°C, washed in cold PBS and then harvested by scraping. Cell pellets were then re-suspended in RIPA lysis buffer (NaCl 150 mM, Nonidet P-40 1%, Sodium deoxycholate 0.5%, Sodium dodecyl-sulphate 0.1%, TRIS 25 mM, pH 7.4) containing protease inhibitor cocktail at the concentration suggested by the manufacturer, sonicated for 2×10 seconds at 4°C with UP 200s ultrasonic device at the at maximum power (Hielsher Ultrasonics, Teltow, Germany) and then maintained under gentile shaking for further 30 minutes at 4°C.

Subcellular fractionation was obtained by subcellular protein fractionation kit according to the manufacturers’ procedures (Thermo Scientific Inc, Rockford, IL). Stepwise isolation of cellular compartments from average 5×10^6^ cells was performed after the application of the supplied cytoplasmic and membrane protein extraction buffers, in order to address protein localization and redistribution studies (16). The protein concentration in was estimated using the BCA Protein assay kit (Abcam, Cambridge, UK) and the indicated amount of protein was subjected to western blotting.

Protein samples were denatured by boiling for 10 minutes in loading buffer containing sodium dodecyl sulphate and 2-β-mercaptoethanol, and then fractionated using SDS-PAGE gel (Bio-Rad). After blotting onto Nitrocellulose Membrane (Bio-Rad, Milano Italy) and blocking with 5% nonfat milk in 0.1% Tween 20/PBS buffer, blots were incubated overnight at 4°C with the primary antibody (all diluted 1:1000) whose immunoreaction was detected by the incubation with a properly diluted HRP-conjugated secondary antibody (1:1000, Cell Signaling Tech.) and visualized with Chemiluminescent Substrate LumiGLO Reserve (Seracare, Milford, MA, USA). Signals were acquired with the Chemidoc XRS System (Bio-Rad). β-Actin (Abcam) served as internal control.

### Docking Analysis

The possible association of PFOA and GM3 molecules with human InsR was theoretically investigated by docking methods. To perform the analysis, the starting point is usually the tertiary structure of the involved molecules. The 3D model of perfluorooctanoic acid (PFOA, C8HF15O2) was retrieved from the PubChem database (https://pubchem.ncbi.nlm.nih.gov; PubChem CID: 9554) and the transmembrane domain of human IR was obtained from the Protein Data Bank (https://www.rcsb.org; PDB code: 2MFR). From the Protein Data Bank the model of the glicolipid transfer protein with bound GM3 ganglioside (PDB code: 2BV7) was also retrieved to obtain a GM3 structure that was further refined by energy minimization (https://www.yasara.org/minimizationserver.htm) (17) in a lipid environment of phosphatidylcholine prepared using MemGen (http://memgen.uni-goettingen.de) (13, 18).

Chosen structures were prepared for docking according to standard procedures. All extra molecules such as ligands or additional subunits were first removed from the receptor model. Then, all the molecules of interest (receptor and possible ligands) underwent processing steps including deletion of water molecules, repairing of truncated chains, adding of hydrogens and partial charge assignment. The obtained structures were finally stored for further processing. All these calculations were performed by using the *DockPrep* module, available in the *UCSF Chimera* molecular modelling software (Resource for Biocomputing, Visualization, and Informatics, University of California, San Francisco; https://www.rbvi.ucsf.edu/chimera). Docking of ligands to the receptor was then performed by using the *AutoDock Vina* software (19). The predicted molecular complexes were scored according to Gibbs free energy of binding and RMSD values, and for each studied ligand the docking solution with the best scores was considered. Following the same approach, docking of GM3 to the complex formed by InR and PFOA was also analyzed. A theoretical estimate of the binding affinities was obtained by using the PRODIGY tool (https://bianca.science.uu.nl/prodigy/) (20).

### Statistical Analysis

Statistical analysis of the data was conducted with SPSS 21.0 for Windows (SPSS, Chicago, IL, USA). The comparison between two groups of data, obtained from western blot analysis and cytochemical staining, were determined by paired two-tailed Student’s t-test after acceptance of normal distribution of the data with the Kolmogorov–Smirnov test. One-way ANOVA with Bonferroni correction was used for the comparison of more than 2 groups of data. Values of P<0.05 were considered as statistically significant

## Results

### Short Term Exposure to PFOA Alters Glycogen Synthesis and Glucose Uptake in HepG2 Cells

**Figure 1:**
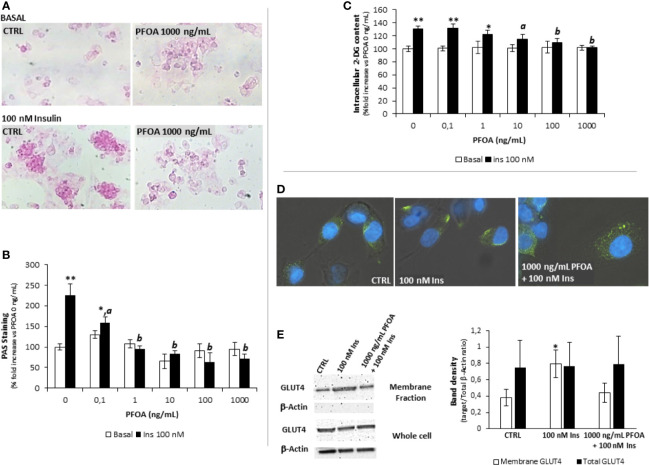
**(A)** Representative images of Periodic Acid Schiff (PAS) staining in HepG2 cells, in basal condition and after stimulation with 100 nm insulin (Ins), exposed for 24h to perfluoro-octanoic acid (PFOA) at the concentration of 0 (CTRL) and 1000 mg/mL. The histogram plot in **(B)** shows the corresponding results of image analysis on PAS staining intensity. Data are reported as the percentage fold increase compared to control condition, in which both PFOA and Ins were omitted, and represent the mean value of three independent experiments. Significance: *P<0,05 and **P < 0,01 respectively *vs* corresponding basal where Ins was omitted. aP < 0 ,05 and bP < 0,01 *vs* control + 100 nm Ins. **(C)** Evaluation of glucose uptake in HepG2 cells by the 2-deoxyglucose (2-DG) assay. Intracellular 2-DG was quantified in basal condition and after stimulation with 100 nm insulin, after the exposure for 24h to PFAS at increasing concentration. Data are reported as the percentage fold increase compared to control condition, in which both PFOA and Ins were omitted, and represent the mean value of a quadruplicate. Significance: *P < 0,05 and **P < 0,01 respectively *vs* corresponding basal where Ins was omitted. aP < 0,05 and bP < 0,01 *vs* control + 100 nm Ins. **(D)** Representative images of immunofluorescence assay for Glut-4 glucose transporter (green) in HepG2 cells at basal (CTRL) and stimulated with 100 nm Insulin with or without previous exposure to PFOA at the concentration of 1000 ng/mL. Cells were counterstained with DAPI (blue) and merged with bright-field images. Pictures are representative of three independent experiments. **(E)** Representative images of Western Blot analysis for Glut-4 protein expression in HepG2 cells at basal (CTRL) and stimulated with 100 nm Insulin with or without previous exposure to PFOA at the concentration of 1000 ng/mL, in the whole cell extract and after membrane protein fractionation. β-Actin in the whole cell extract was used as housekeeping. The histogram reports the target band density/total β-Actin density ratio for Glut-4 in the membrane fraction and whole cell extract. Data represent the mean value of three independent experiments. Significance: *P < 0.05 *vs* CTRL.

The possible effect of the short term exposure to PFOA on glucose metabolism in hepatic cells was initially investigated through the evaluation of glycogen synthesis under Ins stimulation. To this end, starved HepG2 cells were exposed for 24 hours to PFOA, at concentrations ranging from 0 ng/mL (control) to 1000 ng/mL, and then stimulated with 100 nM Ins. The extent of glycogen synthesis was assessed by PAS staining as previously described (21) and representative images are reported in **Figure 1A**. As expected, in control condition Ins stimulation was associated with a marked increase of PAS staining compared to unstimulated basal samples. However, exposure to increasing concentration of PFOA was associated with a progressive weakening of the staining signal in Ins stimulated samples compared to corresponding basal. Image analysis of PAS staining intensity showed that even at the lowest concentration of PFOA tested (0,1 ng/mL), the PAS staining upon Ins stimulation was markedly reduced compared to control condition (respectively: 225,3 ± 27,5% control+ Ins *vs* 158,6 ± 14,4% PFOA 0,1 g/mL + Ins; P=0.048; **Figure 1B**). The exposure to PFPA at a concentration equal or greater than 1 ng/mL was associated with non-significant increase of the PAS staining in cells stimulated with Ins compared to corresponding unstimulated basal condition.

On the base of the observed disorders in glycogen synthesis associated with the exposure to PFOA, the possible impairment of glucose uptake was then evaluated. Accordingly, starved HepG2 cells were exposed for 24 hours to PFOA at increasing concentration and the extent of glucose uptake under Ins stimulation was evaluated through the 2-DG assay (22). HepG2 cells were fully competent in up-taking extracellular glucose upon Ins stimulation, as observed by the significant increase of intracellular 2-DG in control condition (respectively 100,0 ± 4,2% basal control *vs* 130,6 ± 4,5% control + Ins; P=0,001). However, the exposure to PFOA at concentration of 10 ng/mL was associated with a significant reduction of intracellular 2-DG levels upon Ins stimulation (115,0 ± 6,9% PFOA 10 ng/mL + Ins; P=0,031 *vs* control + Ins). At concentration of PFOA greater than 10 ng/mL, Ins stimulation was associated with non-significant increase of intracellular 2-DG content compared to the corresponding basal condition in which Ins stimulation was omitted (**Figure 1C**).

In parallel with the impairment of glucose uptake, the immunofluorescence assay showed that, upon Ins stimulation, the exposure to PFOA at the highest concentration tested of 1000 ng/mL was associated with the persistence of the immune-staining for Glut-4 glucose transporter within the cell cytoplasm, contrarily to control condition, in absence of PFOA, where Ins stimulation was clearly associated with the detection of Glut-4 signal essentially on cell membrane (**Figure 1D**). In order to confirm the possible impairment of Glut-4 translocation on cell membrane upon Ins stimulation, subcellular protein fractionation was performed, by the use of a specific subcellular protein fractionation kit, and the expression of Glut-4 on the cell membrane fraction was detected by western blot analysis (**Figure 1E**). The complete lack of signal for β-Actin on the plasma membrane protein fraction confirmed the effectiveness of the fractionation process. The comparison of the protein band density showed that, that in spite of an unchanged total protein expression of Glut-4 on the whole cell extract, the portion translocated on the plasma membrane upon the stimulation with Insulin was nearly halved by the exposure to PFOA and non-significantly different from the control (respectively, reported as target/Total b-Actin ratio: CTRL 0.38 ± 0.10; 100 nM Ins 0.79 ± 0.17 P=0.0223 *vs* CTRL; 1000 ng/mL PFOA + 100 nM Ins 0.44 ± 0.12 P=0.511 *vs* CTRL).

Taken together, these evidence of major impairment of glucose uptake and glycogen synthesis associated with the exposure to PFOA upon insulin stimulation are suggestive of a possible impact of this PFAS on signaling pathway mediated by InsR in human hepatocyte cell line.

### Short Term Exposure to PFOA Associates With an Early Uncoupling the Insulin Receptor Activation From the Downstream Signaling Pathway

**Figure 2:**
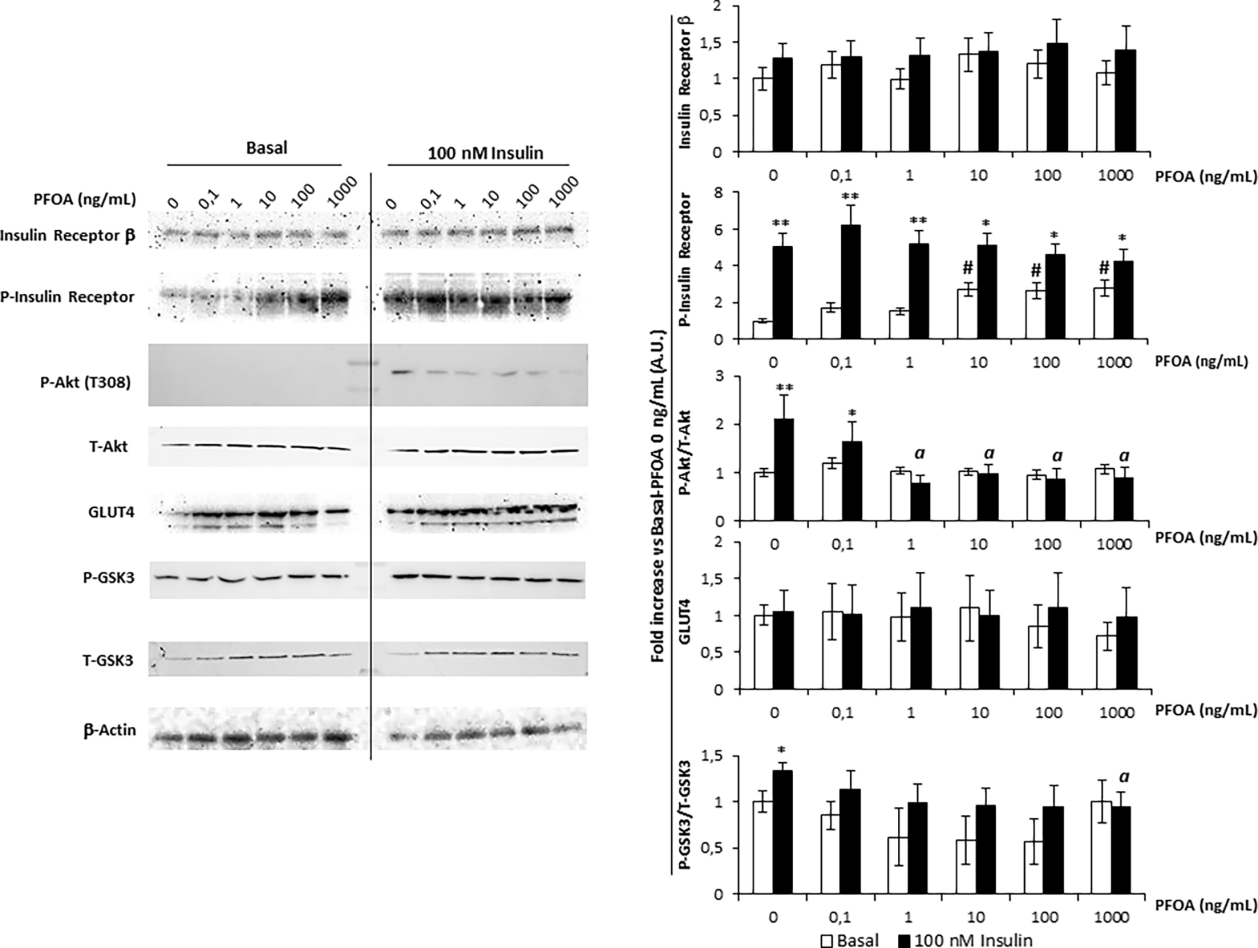
Western blot analysis of Insulin Receptor signaling pathway in HepG2 cells, in basal condition and stimulated with 100 nm Insulin, after the exposure for 24 with perfluoro-octanoic acid (PFOA) at increasing concentration. β-Actin served as housekeeping. The histogram plot in shows the corresponding results of band density analysis. Data were normalized on the corresponding β-Actin and reported as the fold increase compared to control condition, in which both PFOA and Ins were omitted, representing the mean value of three independent experiments. Significance: *P < 0,05 and **P < 0,01 respectively *vs* corresponding basal where Ins was omitted. ^a^P < 0,05 *vs* control + 100 nm Ins. ^#^P < 0,05 *vs* control condition in which both PFOA and Ins were omitted.

In order to disclose the possible impact of PFOA on Ins signaling in hepatocytes, HepG2 were exposed to the perfluoro-alkyl chemical for 24 hours at increasing concentration from 0 ng/mL (control) to 1000 ng/mL. Cells were then pulsed with 100 nM Ins for 20 minutes and the effect on the downstream pathway of InsR was evaluated by western blot analysis (**Figure 2**).

Importantly, the expression of InsR appeared to be unaffected by the exposure to PFOA at any of the concentration tested. The evaluation of P-InR levels was then performed in order to assess the tyrosine-kinase activity which is necessary for InsR auto-phosphorylation and activation (23). Curiously, exposure to PFOA at a concentration equal or greater than 10 ng/mL was associated with a significant increase of P-InsR levels even in basal condition in absence of Ins Stimulation. However, tyrosine-kinase activity of InR showed to be fully functional since the stimulation with Ins was associated with a significant increase of P-InsR levels compared to the corresponding basal condition, regardless of the exposure to PFOA.

On the other hand, the downstream activation of InsR signaling appeared to be significantly impaired by the exposure to PFOA since, with the increase of its concentration. In fact, the P-Akt/T-Akt ratio upon Ins stimulation showed a progressive decline compared to control condition in which PFOA was omitted. In particular, at a concentration of PFOA equal or greater than 1 ng/mL, the P-Akt/T-Akt ratio upon Ins stimulation showed to be significantly lower than the control stimulated with Ins (2,2 ± 0,5 A.U. control + Ins *vs* 0,8 ± 0,2 A.U. PFOA 1 ng/mL + Ins, P=0.0108).

The exposure to PFOA showed also to have an impact on GSK3 phosphorylation, the downstream target of P-Akt. In fact, Ins stimulation after the exposure to PFOA from the concentration of 0.1 ng/mL onward, showed to be ineffective to increase the P-GSK3/T-GSK3 ratio compared to the corresponding unstimulated sample. In addition, after the exposure to PFOA at the concentration of 1000ng/mL, the P-GSK3/T-GSK3 ratio upon Ins stimulation was significantly lower than that observed in control condition where no exposure to PFOA was applied.

Considering that neither the exposure to PFOA nor the stimulation with Ins was associated with variations in Glut-4 expression, taken together these data suggest that the reduced glucose uptake and intracellular glycogen synthesis associated with the exposure to PFOA are not related to defective InR or Glut-4 expression but rather to an upstream uncoupling between InR activation and Akt phosphorylation.

### Docking Analysis Shows That the Exposure to PFOA Likely Uncouples Early Insulin Signaling by Stabilizing the Interaction Between Insulin Receptor Gangliside GM3

Sensitivity to Ins has been demonstrated to largely depend on several cell factors, including the membrane composition. In particular, the insulin resistance featuring obesity has been related to the low grade chronic inflammation which, in turn, associates with the overexpression of TNF-α in adipose tissue (24). Increased levels of TNF-α resulted in modified membrane composition of the adipocyte, such as the increased representation of the monosialo-dihexosyl-ganglioside, known as GM3 (25). According to the model proposed by Inokuchi (26), in adipocytes rendered insulin resistant by treatment with TNF-α, InsR accumulates in GM3-enriched membrane microdomains, weakening of downstream signaling pathway although both the expression of InsR and its auto-phosphorylation activity remain unaltered. On this base, we asked whether the apparent uncoupling between InR activation and the downstream signaling pathway following the exposure to PFOA may be related with the interaction between InsR and GM3 in membrane microdomains.

**Figure 3:**
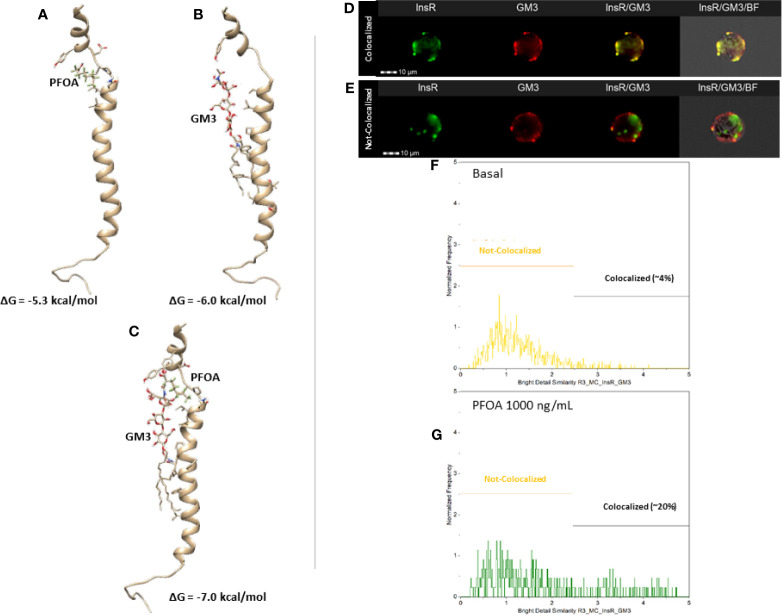
Representative results of the computational docking analysis of perfluoro-octanoic acid (PFOA, **A**), monosialo-dihexosyl-ganglioside (GM3, **B**) and both **(C)** on insulin receptor β-chain. The estimated free Gibbs energy variation (ΔG) values associated with the formation of each complex are reported. Staining pattern of Insulin Receptor (InsR, green) and GM3 ganglioside (GM3, red) in HepG2 at basal **(D)** and after 24 hours exposure to PFOA 1000 ng/mL **(E)** at Flow Cytometry Imaging analysis. The respective the distribution of the staining co-localization of the two antigens is reported in the histogram plot **(F, G)** Data are representative of three independent experiments.

Data from docking simulations suggested that both PFOA and GM3 can bind to IR, as expressed by their Gibbs free energy (ΔG) changes upon binding of about -5.3 kcal/mol and -6.0 kcal/mol (**Figures 3A, B**, respectively). Interestingly, when the interaction between GM3 and the IR-PFOA complex was investigated, the affinity exhibited by the ganglioside resulted even higher, being the predicted ΔG of about -7.0 kcal/mol (**Figure 3C**). These computational data suggest that the exposure to PFOA might borrow the increased production of GM3 due to inflammatory state by stabilizing the interaction between InsR and GM3 in membrane microdomains, possibly explaining the subsequent uncoupling from the subsequent downstream signaling events.

In order to provide a confirm of this mechanistic hypothesis, an imaging flow cytometry approach was adopted to investigate the possible variation of InsR and GM3 localization pattern associated with the exposure to PFOA 1000 ng/mL for 24h. The software-assisted image analysis was clearly able to identify two cell populations characterized by the colocalized and not-colocalized staining of InsR and GM3, respectively (**Figures 3D, E**). The analysis of the distribution of the two staining pattern showed that the exposure to PFOA nearly fivefold the percentage of cells with clear co-localization of the staining for InsR and GM3 (**Figures 3F, G**. Respectively: 3.8 ± 2.6% Basal; 20.2 ± 5.8% PFOA 1000ng/mL; P=0.0111).

### Prolonged Treatment With Glucosyl-Ceramide Synthase Inhibitor Restores Insulin Signaling in HepG2 Cells Exposed to PFOA

**Figure 4:**
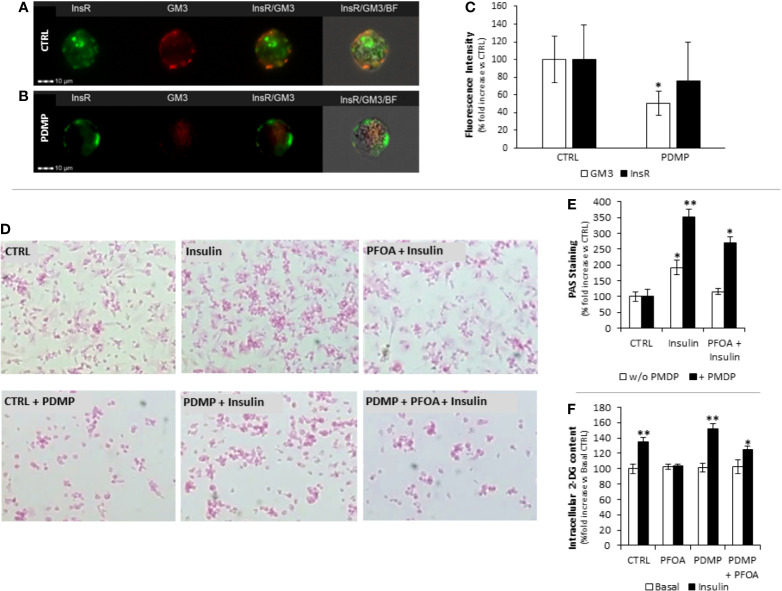
Staining pattern of Insulin Receptor (InsR, green) and GM3 ganglioside (GM3, red) in HepG2 at basal (CTRL, **A**) and after 96 hours treatment with (±)-threo-1-Phenyl-2-decanoylamino-3-morpholino-1-propanol hydrochloride (PDMP, **B**) 20 ng/mL at Flow Cytometry Imaging analysis. In **(C)** the evaluation of the fluorescence intensity is reported as percentage fold increase compared to control. Data report the mean value of three independent experiments. Significance: *P < 0.05 *vs* CTRL. **(D)** Representative images of Periodic Acid Schiff (PAS) staining in HepG2 cells, in basal condition (CTRL) and after stimulation with 100 nm insulin (Ins), exposed for 24h to perfluoro-octanoic acid (PFOA) at the concentration of 0 ng/mL (CTRL) and eventually treated for 96 hours with PDMP. The histogram plot in **(E)** shows the corresponding results of image analysis on PAS staining intensity. Data are reported as the percentage fold increase compared to the corresponding control condition, in which both PFOA and Ins were omitted, and represent the mean value of three independent experiments. Significance: *P < 0,05 and **P < 0,01 respectively *vs* corresponding control. **(F)** Evaluation of glucose uptake in HepG2 cells by the 2-deoxyglucose (2-DG) assay. Intracellular 2-DG was quantified in basal condition and after stimulation with 100 nm insulin, after the exposure for 24h to 1000 ng/mL PFOA and eventually treated for 96 hours with PDMP. Data are reported as the percentage fold increase compared to control condition, in which both PFOA and Ins were omitted, and represent the mean value of a quadruplicate. Significance: *P < 0,05 and **P < 0,01 respectively *vs* corresponding basal where Ins was omitted.

In order to confirm the role of GM3 in the impairment of InsR signaling associated with PFOA exposure, we depleted the cell content of gangliosides by treating HepG2 cells with PDMP, a known inhibitor of glucosyl-ceramide synthase used to investigate the functional role gangliosides in cell biology (27). The treatment of HepG2 cells for 96 hours with 20 μM PDMP showed a significant reduction of the staining intensity for GM3 evaluated by imaging flow cytometry, but no significant effect was observed for InsR (**Figures 4A–C**). On these bases, cells were treated for 96 hours with 20 μM PDMP and, during the last 24 hours of which, cells exposed to PFOA at the highest concentration tested of 1000 ng/mL. Cells were then stimulated with 100 nM Ins and the extent of glycogen synthesis was assessed by PAS staining (**Figure 4D**). Interestingly, the treatment with PDMP by itself was associated with a marked increase of glycogen accumulation, compared to untreated cells, after Ins stimulation (191,7 ± 23,0% control + Ins *vs* 351,2 ± 25,9% PDMP + Ins; P=0,0042). Furthermore, the impaired glycogen synthesis associated with the exposure to PFOA was largely restored by the long term treatment with PDMP (115,4 ± 9,6% PFOA + Ins *vs* 268,9 ± 21,2% PDMP + PFOA + Ins; P<0,001 **Figure 4E**). The possible effect of PDMP treatment on the impaired glucose uptake associated with the exposure to PFOA was also investigated by the use of the 2-DG assay (**Figure 4F**). The sole long-term treatment with PDMP was able to increase intracellular 2-DG in HepG2 cells stimulated with Ins, compared to untreated cell (134,6 ± 5,5% control + Ins *vs* 152,2 ± 6,2% PDMP + Ins; P=0.042). On the other hand, intracellular 2-DG levels upon Ins stimulation in HepG2 cells exposed to PFOA were significantly increased by the long term treatment with PDMP, compared to untreated controls (103,4 ± 2,5% PFOA + Ins *vs* 125,5% ± 3,8% PDMP + PFOA + Ins; P=0,041).

**Figure 5:**
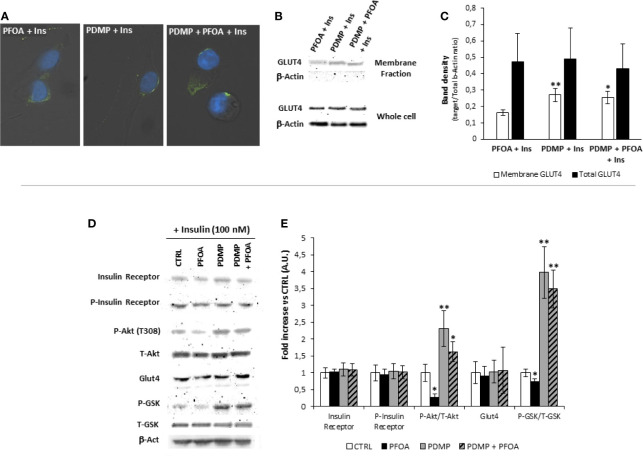
**(A)** Representative images of immunofluorescence assay for Glut-4 glucose transporter (green) in HepG2 cells stimulated with 100 nm Insulin, with or without previous exposure to PFOA at the concentration of 1000 ng/mL, and eventually treated for 96 hours with (±)-threo-1-Phenyl-2-decanoylamino-3-morpholino-1-propanol hydrochloride (PDMP) 20 ng/mL. Cells were counterstained with DAPI (blue) and merged with bright-field images. Pictures are representative of three independent experiments. **(B)** Representative images of Western Blot analysis for Glut-4 protein expression in HepG2 cells at basal (CTRL) and stimulated with 100 nm Insulin with or without previous exposure to PFOA at the concentration of 1000 ng/mL, in the whole cell extract and after membrane protein fractionation. β-Actin in the whole cell extract was used as housekeeping. The histogram in **(C)** reports the target band density/total β-Actin density ratio for Glut-4 in the membrane fraction and whole cell extract. Data represent the mean value of three independent experiments. Significance: *P < 0.05 *vs* CTRL. **(D)** Western blot analysis of Insulin Receptor signaling pathway in HepG2 cells stimulated with 100 nm Insulin, after the exposure for 24 with PFOA at the concentration of 1000 ng/mL and eventually treated for 96 hours with PDMP. β-Actin served as housekeeping. The histogram plot in **(E)** shows the corresponding results of band density analysis. Data were normalized on the corresponding β-Actin and reported as the fold increase compared to control condition, in which PFOA was omitted, representing the mean value of three independent experiments. Significance: *P < 0,05 and **P < 0,01 respectively *vs* corresponding control stimulated with insulin.

From a mechanistic point of view, the treatment with PDMP was associated with a partial recovery of Glut-4 translocation to cell membrane upon Ins stimulation as observed by immunofluorescence assay (**Figure 5A**).

In order to confirm the possible impairment of Glut-4 translocation on cell membrane upon Ins stimulation, was performed, by the use of a specific subcellular protein fractionation kit, and (**Figures 5B, C**). The complete lack of signal for β-Actin on the plasma membrane protein fraction confirmed the effectiveness of the fractionation process. The comparison of the protein band density showed that, that in spite of an unchanged total protein expression of Glut-4 on the whole cell extract, the treatment with PDMP was able to significantly increase the signal for Glut-4 on the membrane protein fraction even when the exposure to PFOA 1000 ng/mL was applied (respectively, reported as target/Total b-Actin ratio: 0.16 ± 0.02 PFOA 1000 ng/mL + Ins 100 nM; 0.26 ± 0.04 PDMP + PFOA 1000 ng/mL + Ins 100 nM; P= 0.018).

From a molecular point of view, western blot analysis of the InsR signaling pathway performed on HepG2 cells stimulated with 100 nM Ins, showed that long term treatment with PDMP had no major effect on either InsR, or its auto-phosphorylation activity, as well as on the overall Glut-4 expression (**Figures 5D, E**). However cell ganglioside depletion obtained by the use of glucosyl-ceramide synthase inhibitor was able to significantly increase both P-Akt/T-Akt ratio and P-GSK3/T-GSK3 ratio upon Ins stimulation, and to largely restore these signaling events that appeared reduced by the exposure to PFOA, compared to unexposed control.

## Discussion

By the use of an *in vitro* approach, in the present study we provide evidence that the acute exposure of human hepatocellular carcinoma cell line HepG2 to perfluoro-octanoic acid associates with the impairment of insulin receptor signaling, resulting in reduced glucose uptake and reduced glycogen synthesis. From a mechanistic point of view, this effect likely relies on the interference of PFOA with the InsR at membrane level, resulting early uncoupling between InR activation/autophosphorylation and downstream Akt and GSK3 phosphorylation. Importantly, the docking analysis suggests that PFOA might weaken the Ins stimulus by stabilizing the interaction between InsR and specific gangliosides components within membrane microdomains, with the consequent early disturbance of InsR activity. This hypothesis was confirmed by the large recovery of InsR signaling, glucose uptake and glycogen synthesis obtained in HepG2 cells exposed to PFOA after the depletion of gangliosides by the use of a known glucosyl-ceramide synthase inhibitor. To the best of our knowledge, this is the first study linking the altered hepatocyte function to PFAS exposure without involving the tissue accumulation resulting from prolonged exposure.

Incident diabetes and increased fasting glucose have been variably described as clinical outcomes associated with PFAS exposure, largely depending on the molecule type, level of exposure, age, underlying comorbidities, developmental period of exposure and geographic area (28–32). However, diabetes-related cardiovascular mortality have been recently claimed as major complication associated with environmental exposure to PFAS, particularly in the Veneto Region of Italy where the exposure to PFOA, deriving from anthropogenic contamination of industrial origin, has proved to be an important public health issue (4, 33). On these bases, given both the key role of the liver in energy metabolism and the reported high tissue levels of PFOA in this organ, a pathogenic role of the pollutant on the altered glucose management by the liver appears plausible. To this regard, the two available studies on this topic were performed in rodent models, in which the animals were equally treated for 28 days with a same dose of 1.25 mg/kg/day of PFOA. Strikingly, the two studies led to almost diametrically opposed conclusions since: the first reported higher insulin sensitivity and glucose tolerance, whilst the second showed increased fasting blood glucose levels and decreased tissue content of glycogen and glucose, respectively (34, 35). It should be noted that a wide range of factors are acknowledged to affect the clinical outcome related to PFAS exposure such as the species of the experimental model used, the gender, the genetic background and the period of exposure by itself (3). Indeed, we recently showed in different experimental models that PFAS in general, and PFOA in particular, are able to exert very early cell function derangements, without necessarily requiring cell-bio accumulation, whose common denominator is the interaction of the contaminant with the plasma membrane (13, 14). On this base, the interaction of these long-lasting chemicals with membranes and cells are likely to lead to unexpected diverse effects depending on the cell identity (7). Accordingly, in this study we report that short-term exposure to PFOA is able to exert significant impairment of the InsR signaling pathway, resulting in downstream reduced glucose uptake and altered glycogen synthesis. Most importantly, our findings point towards a major impact of PFOA on cell membrane architecture/composition such as the stabilization of InsR into ganglioside-rich dicrodomains with the subsequent uncoupling between the receptor activation and the triggering of the downstream signaling events. This is in agreement with a previously described pathogenic model developed to link the low grade chronic inflammation with insulin resistance in adipocytes (36).

While acknowledging the purely experimental nature of this study and the use of the human hepatocellular-carcinoma cell line HepG2, our data reinforce the hypothesis that PFAS toxicity is based on different mechanisms, in addition to the known endocrine interference with steroid hormones (37, 38). Further studies will be needed to clarify the relative weight of this pathogenetic mechanism in determining the clinical outcome of the exposure to PFAS, hopefully in primary human hepatocytes or liver organoids.

In conclusion, by the use of an *in vitro* model, in this study we provide evidence of a pathogenic model linking exposure to PFOA to the development of altered glucose metabolism in liver through the involvement of altered membrane composition and architecture. This model provides a PFOA toxicodynamic mechanism that involves none or poor cell bio-accumulation and represents a suitable approach to study the increasing health consequences associated with the exposure to such class of chemicals.

## Data Availability Statement

The datasets presented in this study can be found in online repositories. The names of the repository/repositories and accession number(s) can be found in the article/supplementary material.

## Author Contributions

LDT, study design, experimental setting and manuscript drafting. ADN, study design and manuscript drafting. MSR, experimental setting and manuscript drafting. DG, computational analysis. ADM, LB, SS, EP and MP, experimental setting. AG, study suprevision and manuscript drafting. CF, study supervision and manuscript drafting. All authors contributed to the article and approved the submitted version.

## Conflict of Interest

The authors declare that the research was conducted in the absence of any commercial or financial relationships that could be construed as a potential conflict of interest.

## Publisher’s Note

All claims expressed in this article are solely those of the authors and do not necessarily represent those of their affiliated organizations, or those of the publisher, the editors and the reviewers. Any product that may be evaluated in this article, or claim that may be made by its manufacturer, is not guaranteed or endorsed by the publisher.
